# Supplementary material for: Shifts in honeybee foraging reveal historical changes in floral resources
Source: Commun Biol. 2021 Jan 14;4:37. doi: 10.1038/s42003-020-01562-4 (PMC7809426; doi:10.1038/s42003-020-01562-4)
Supplement: Supplementary file 2 — Supplementary Information [file 42003_2020_1562_MOESM2_ESM.pdf]

Shifts in honeybee foraging reveal historical changes in floral resources

Laura Jones[1,2], Georgina L. Brennan[3], Abigail Lowe[1,2], Simon Creer[2], Col R. Ford[1], and Natasha de Vere*[1,4].

1. National Botanic Garden of Wales, Llanarthne, SA32 8HG, UK

2. MEFGL, School of Natural Sciences, Bangor University, Bangor, LL57 2UW, UK

3. Centre for Environmental and Climate Research / Aquatic Ecology, Lund University, 223 62 Lund, Sweden

4. IBERS, Aberystwyth University, Aberystwyth, SY23 3FL, UK

Supplementary Information

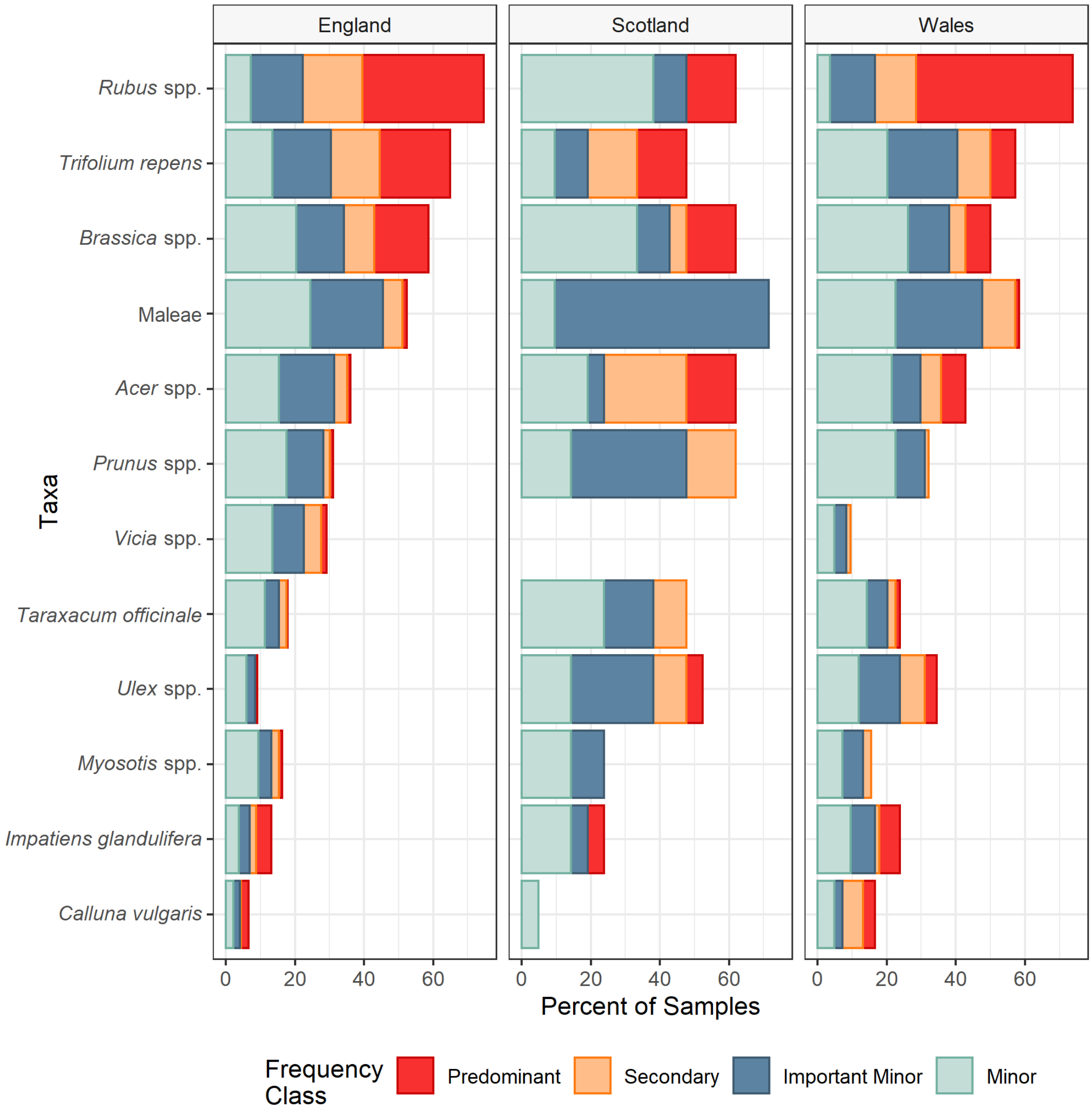

Supplementary Figure 1: Top predominant and secondary taxa (>15% sequences) found in the DNA in the different regions, England (n = 319), Scotland (n = 21), and Wales (n = 84). Regions with smaller samples sizes were excluded: Ireland (n = 3), Northern Ireland (n = 4), Guernsey (n = 3), and the Isle of Man (n = 7).

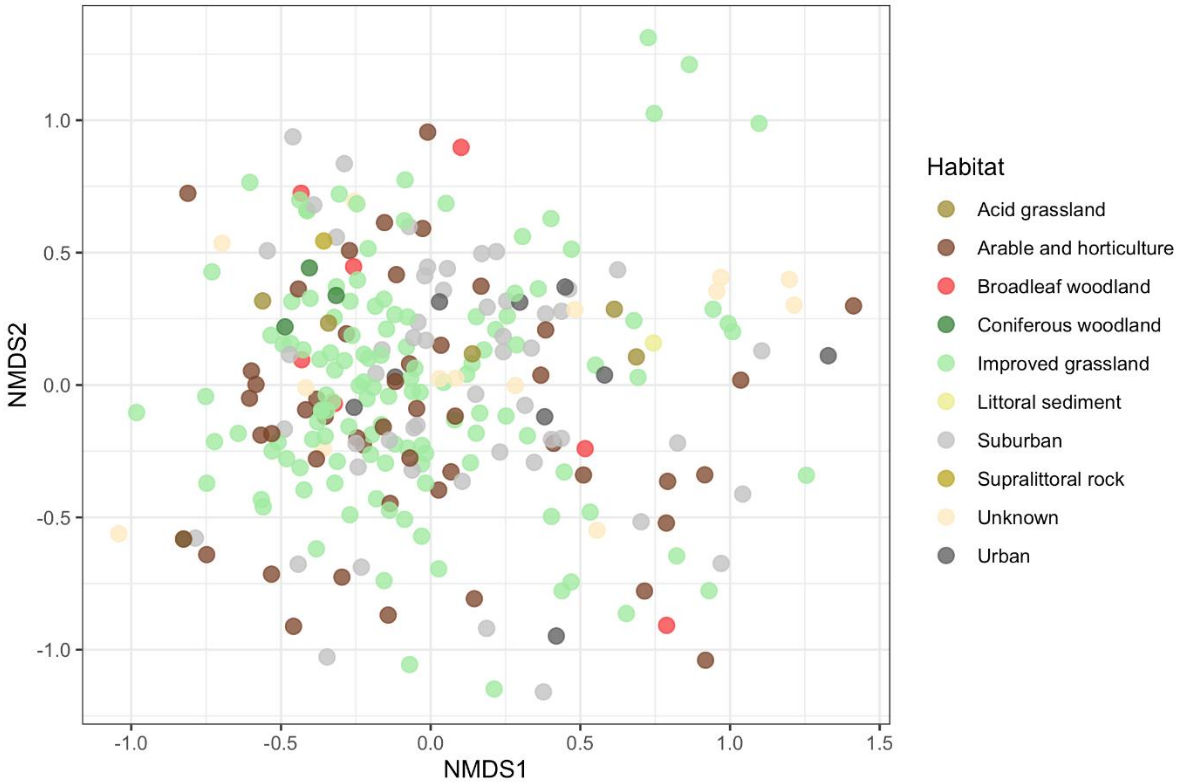

Supplementary Figure 2: Non-metric multidimensional scaling (NMDS) ordination of the 2017 honey samples collected in July and August. Colour indicates the dominant surrounding habitat measured within a 2 km radius of the hive location.

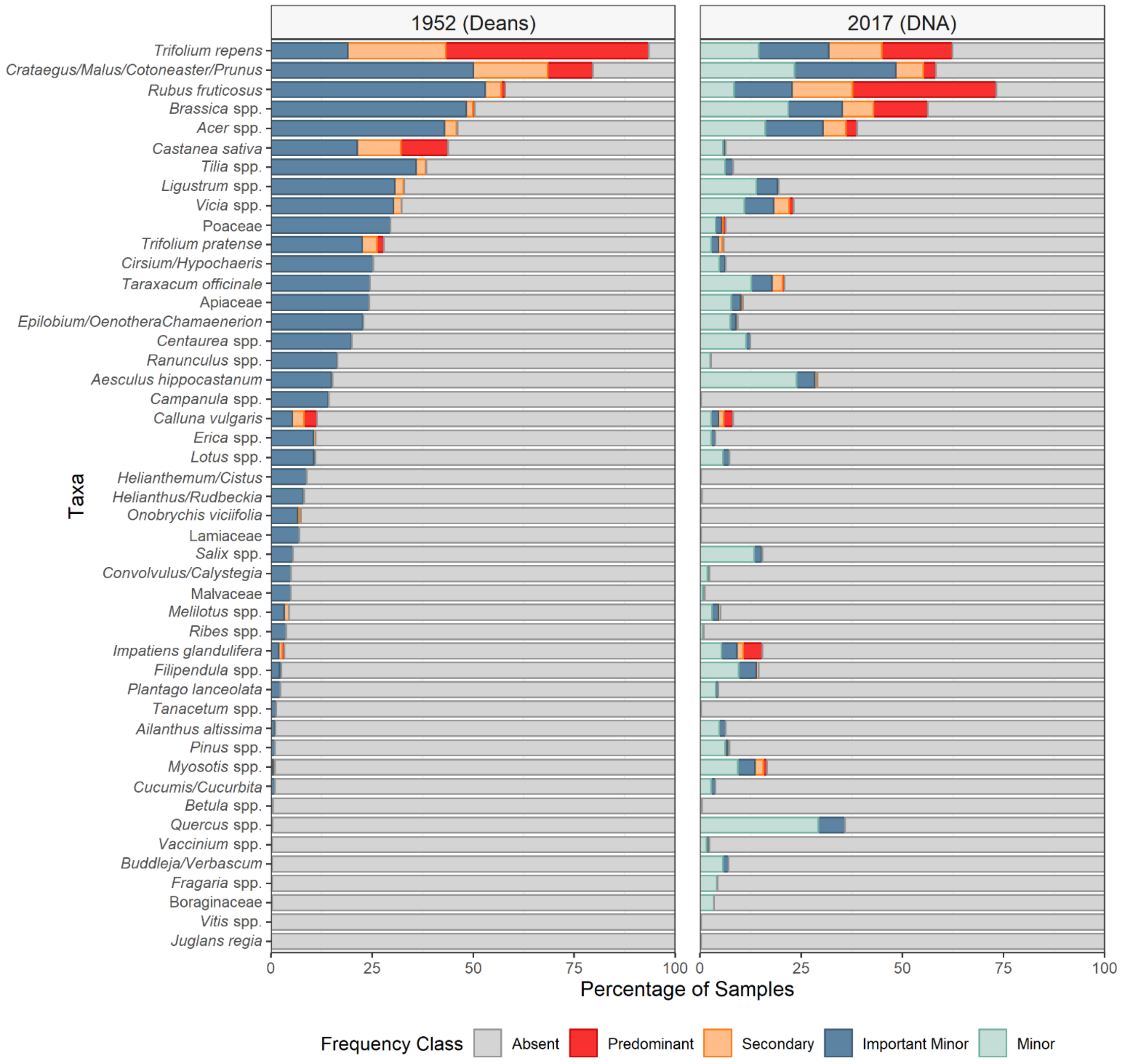

Supplementary Figure 3: Comparing 47 taxa in honey samples analysed by Deans in 1952 (n = 855), using melissopalynology, with honey samples analysed using DNA metabarcoding (n = 441). Overall, there was a positive correlation between the two honey surveys for the total percentage of samples found for each taxa (Kendall's τ correlation coefficient, τ = 0.389, *P* = 0.0001). In order to compare with *the Prunus, Pyrus, Crataegus* group identified by Deans, the DNA reads for the Maleae tribe and *Prunus* were grouped, to create the consensus group of *Crataegus, Malus, Cotoneaster,* and *Prunus.*

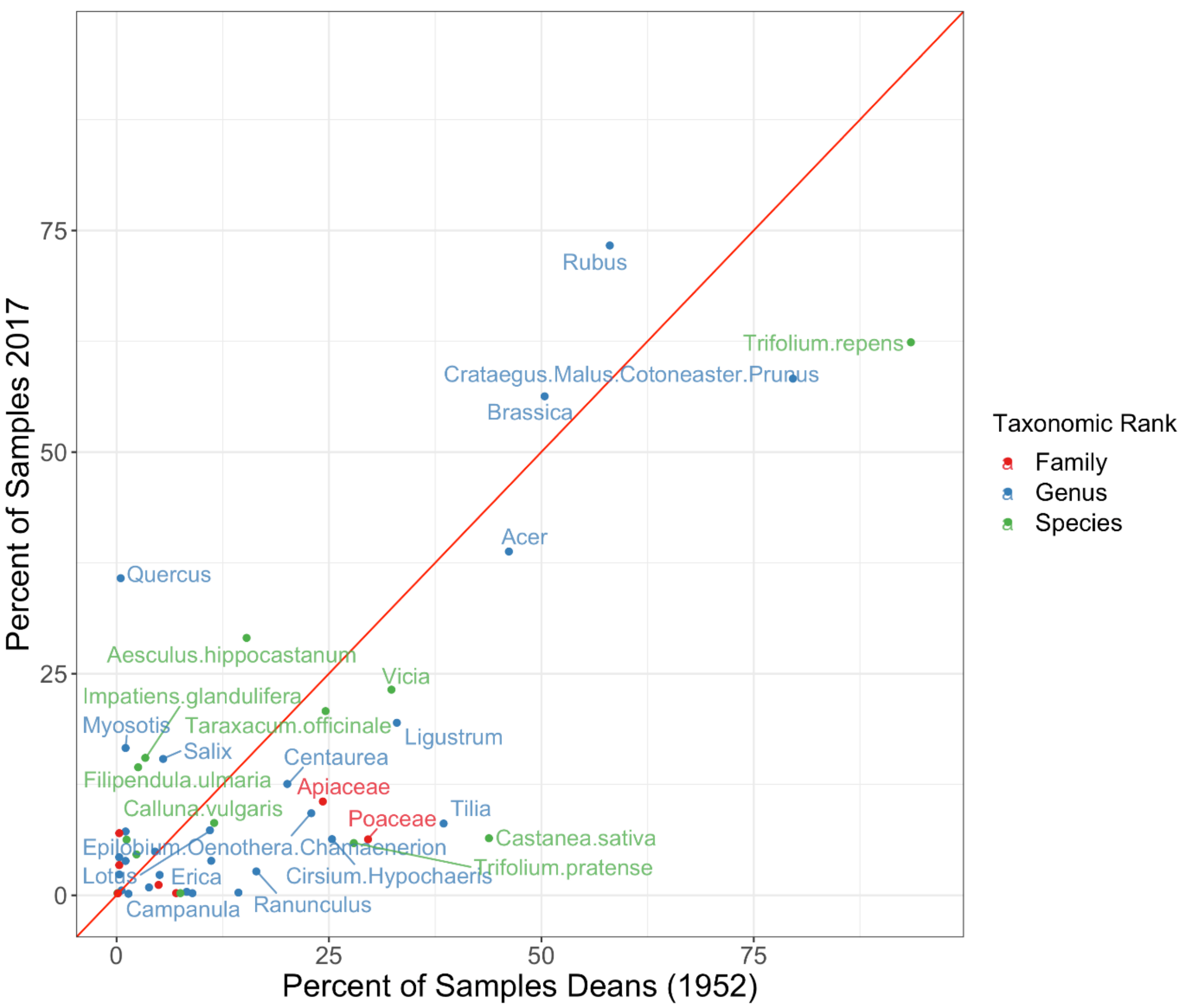

Supplementary Figure 4: Comparing the total proportion of samples found in 1952 and 2017 for the plant taxa found in both surveys. There is a significant positive correlation (Kendall's τ correlation coefficient τ = 0.389, $P < 0.001$). Taxa which appear in over 10% of samples for either the 1952 or 2017 survey are labelled.

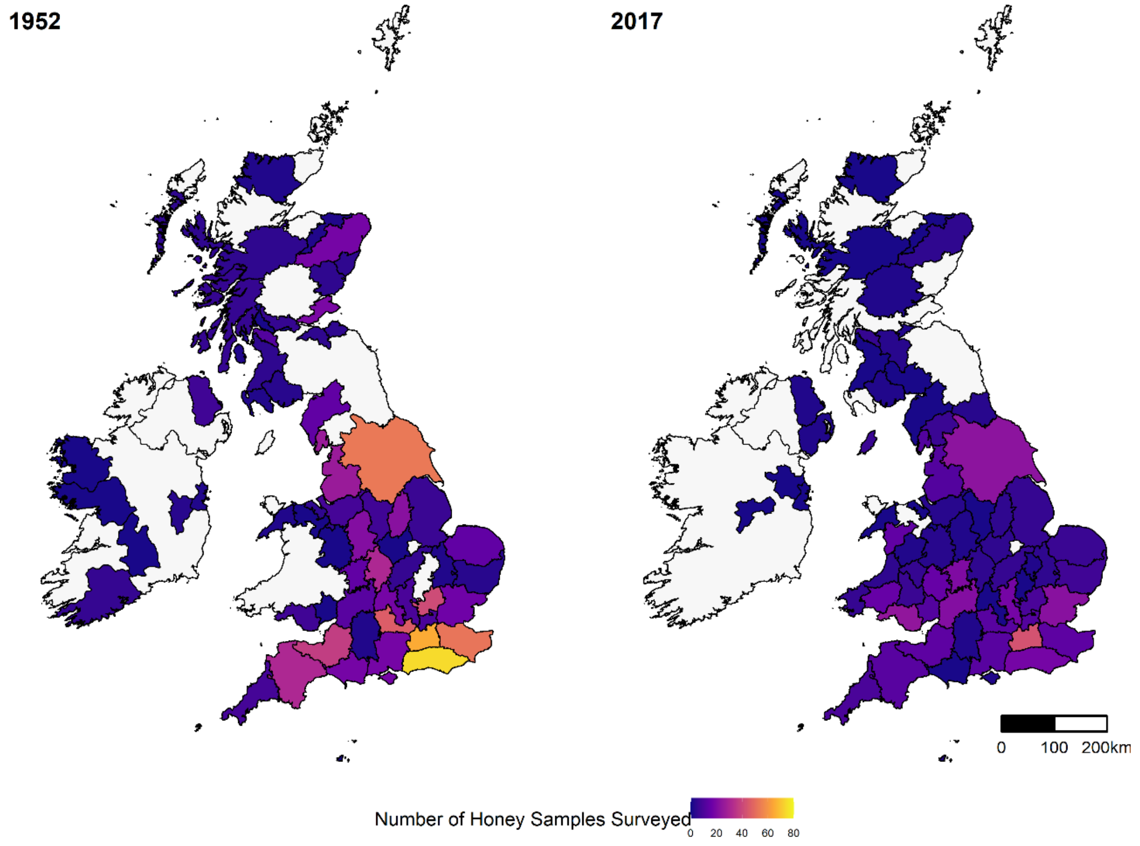

Supplementary Figure 5: A heat map showing the number of honey samples collected from each historical county within the UK and Ireland in 1952 (n = 855) and 2017 (n = 441)**.**

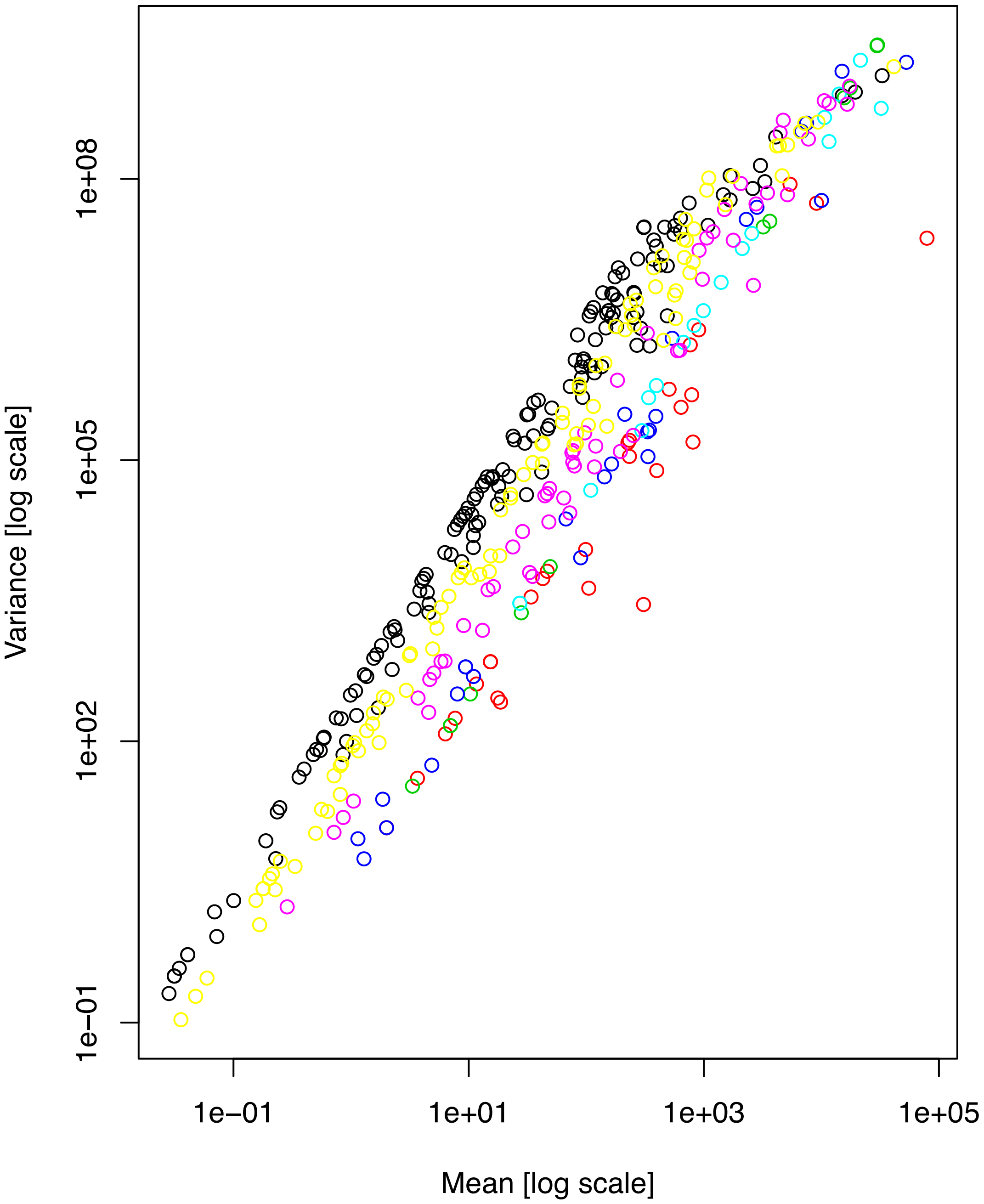

Supplementary Figure 6. There is a strong relationship between the mean proportion of DNA sequences and the variance of the proportion of DNA sequences from each sampling site. Coloured circles denote sampling region. The plots were produced using the meanvar.plot function in the ‘mvabund’ package in R.

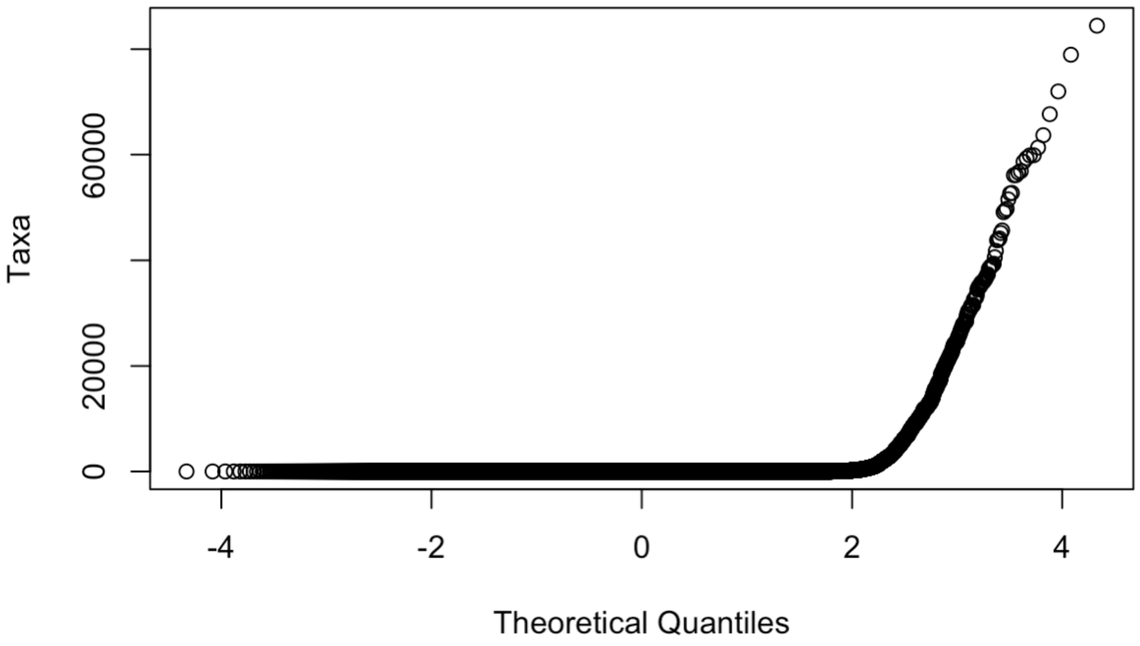

Supplementary Figure 7. Scatter plot of theoretical quantile values taken from a normal distribution and count data produced by metabarcoding (*rbcL* and ITS2 markers). Deviations from a straight line indicate that the count data do not have a normal distribution.

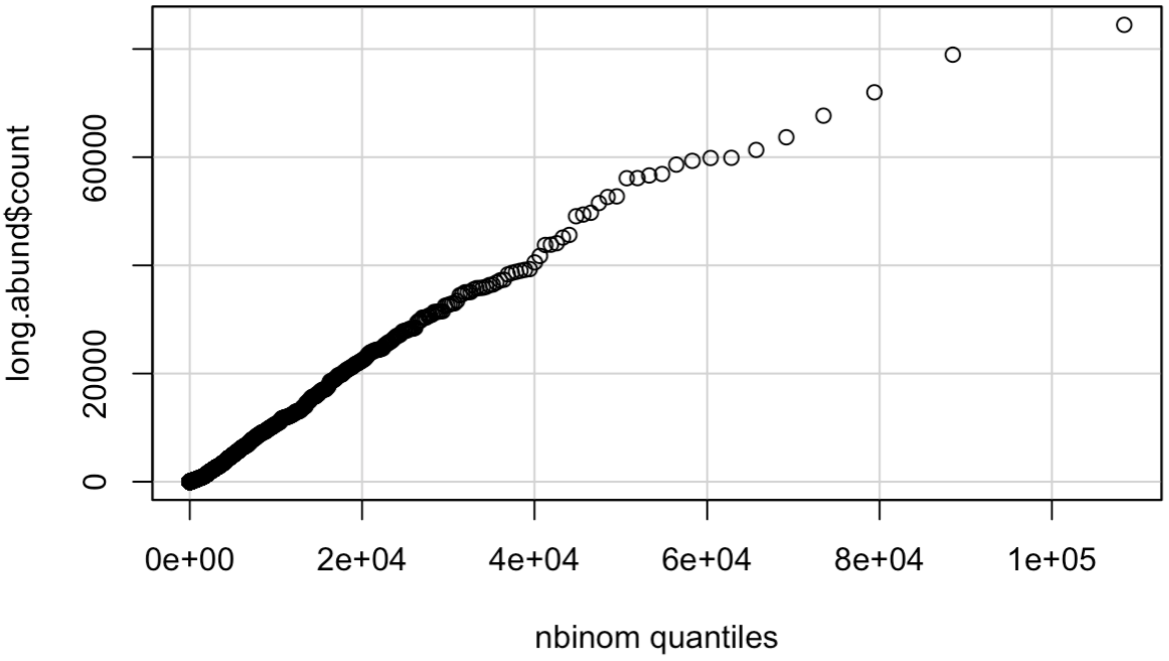

Supplementary Figure 8. Scatter plot of theoretical quantile values taken from a negative binomial distribution and count data produced by metabarcoding (*rbcL* and ITS2 markers). Deviations from the straight line are minimal indicating that the count data have a negative binomial distribution.

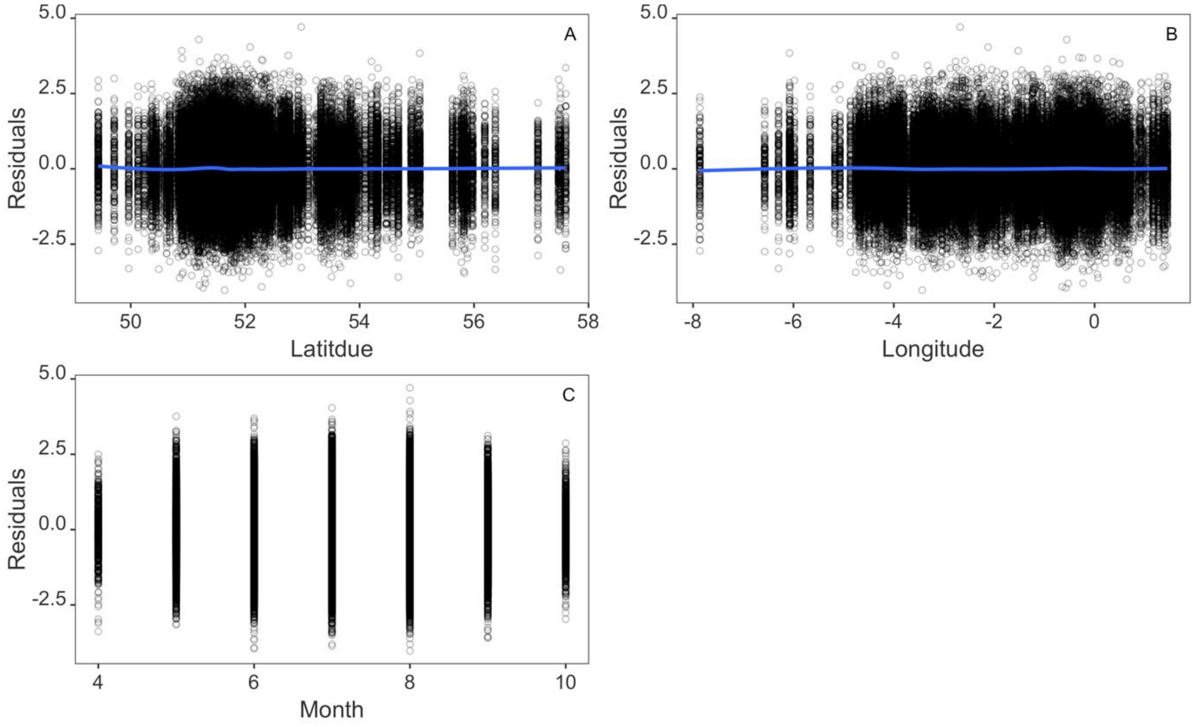

Supplementary Figure 9. Scatter plot of the residuals from the model used to analyse the abundance data produced by metabarcoding (*rbcL* and ITS2 markers) and the model covariates A) Latitute, B) Latitute and C) Time.

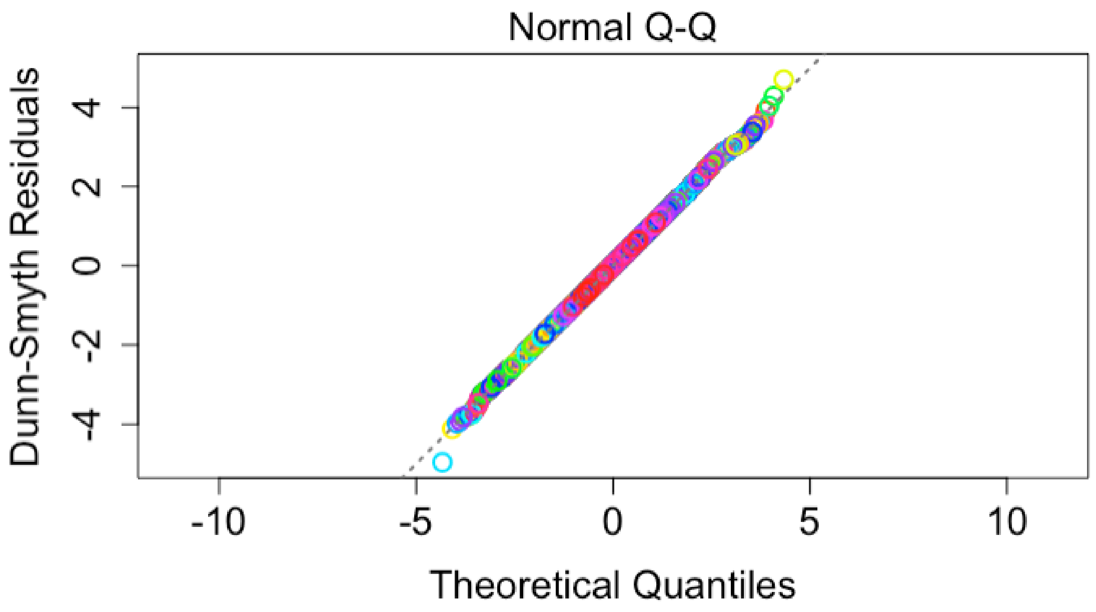

Supplementary Figure 10. Scatter plot of theoretical quantile values and the residuals output from the model used to analyse the abundance data produced by metabarcoding (*rbcL* and ITS2 markers). Deviations from the straight line are minimal indicating a normal distribution and suggests that the model selected is plausible and the mean-variance assumption of the negative binomial regression is correct. Coloured circles denote different genera in the abundance data. The plots were produced using the plot.manyglm function in the mvabund package in R.

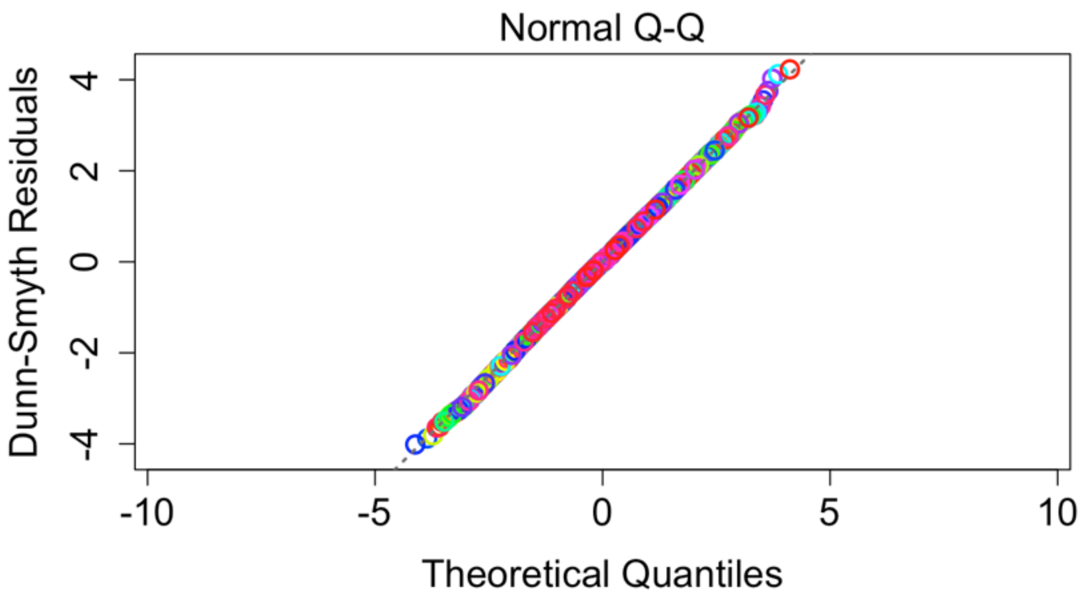

Supplementary Figure 11. Scatter plot of theoretical quantile values and the residuals output from the model used to analyse the abundance data produced by DNA metabarcoding (rbcL and ITS2 markers) and melissopalynology. Deviations from the straight line are minimal indicating a normal distribution and suggests that the model selected is plausible. Coloured circles denote different genera in the abundance data. The plots were produced using the plot.manyglm function in the mvabund package in R.

Supplementary Table 1. Relationship between the presence or absence of insect attractive crop species around the hive and their presence in the honey.

Abundance Class (Percentage of sequencing reads)	*Brassica* spp. with *Brassica napus* present	*Brassica* spp. with *Brassica napus* absent	*Vicia* spp. with *Vicia faba* present	*Vicia* spp. with *Vicia faba* absent
Predominant (>45%)	37	22	5	0
Secondary (15-45%)	25	8	12	5
Important Minor (1-15%)	28	28	22	10
Minor (<1%)	36	58	33	14
Absent	44	138	101	222

The frequency of *Brassica* spp. and *Vicia* spp. found in the 2017 honey samples when their respective crop species, *Brassica napus* and *Vicia faba* were present or absent in a 2 km radius of the hive location. Predominant: >45% of sequences returned in a sample, secondary: 15-45%, important minor: 1-15% and minor <1%.

Supplementary Results

To explore the impact of rarefying the sequencing data to normalise the libraries, the sequencing sampling depth per sample was standardised by using the *rarefy_even_depth* function in the R package phyloseq. The 15th percentile for library size was chosen (n = 8012) and set as the minimum library size, this removed 66 samples from analysis, leaving 375 samples. All of the statistical analyses present in the paper were completed again to examine the conclusions.

The significant relationship between the frequency of the taxa found in both 2017 and 1952 remained (Kendall's τ correlation coefficient, $\tau = 0.371$, $P = 0.0004$). The differences between those plant taxa found abundantly within the honey samples in both surveys (>15% of DNA sequences or pollen grains within the sample) were then examined. The same patterns of increases and decreases in frequency across the honey samples were seen for the nine taxa as presented. All of the statistically significant changes in frequency remained, with the exception of one plant, *Acer*: ($x^2 = 6.853$, d.f = 1, $P = 0.178$).

The spatial relationship between the presence of *Brassica* and *Vicia* crop species in the landscape with their presence in the honey also remained the same. Apiaries with the related crop species present in a 2 km radius of the hives were more likely to have the crop present in the honey for both *Brassica* spp. and oilseed rape (*Brassica napus*) ($x^2 = 45.52$, d.f. = 4, $P < 0.0001$) and *Vicia* spp. with field beans (*Vicia faba*) ($x^2 = 48.13$, d.f. = 4, $P < 0.0001$).

The conclusions from the model examining the effects of time and location were unchanged, with calendar month (April-October) being a good predictor of plant taxa composition (Fig. 2; $LR_{364, 1} = 427.3$, $P = 0.001$). There were no overall regional

differences between England, Scotland and Wales (Supplementary Fig. 1; Latitude $LR_{363,1} = 229.8$, $P = 0.458$; Longitude $LR_{362,1} = 324.8$, $P = 0.195$).

Supplementary Discussion

The results of the honey analysis conducted in 1952[1,2] are supported by other contemporary sources. In England, pollen loads were analysed through the 1945 and 1946 season, with *Trifolium repens* and *Trifolium pratense* identified as the top species found overall[3], while in South Wales *T. repens* and *Rubus fruticosus* were top in pollen loads collected from hives[4]. There were 19 taxa identified in 1952 but not in 2017; nine taxa could not be directly matched to results in the DNA due to differences in taxonomic resolution. For example, in 1952, some of the Asteraceae genera identified (*Tussilago* spp., *Achillea* spp.) may be represented under the family level Asteraceae identification in the DNA. The remaining ten taxa were not represented in the DNA, e.g. *Saxifraga* spp. The taxa missed using the DNA are known to be detected with these primer regions[5,6] and could be explained by the low levels at which they were found in 1952. In 2017, there were an additional 99 taxa not found in 1952 with 15 of these taxa occurring in more than 5% of samples. Similarly, the majority of these taxa were rarely identified at a predominant and secondary abundance (<1%), with the notable exception of *Ulex* spp. (predominant and secondary in 4% of samples). Contemporary sources identify *Ulex* spp. in honeybee pollen loads[3,4], suggesting that this genus was likely missed by Deans[1,2]. Of the 15 taxa found using DNA metabarcoding in over 5% of samples which were not identified by Deans, nine taxa were identified in the pollen loads by Synge (1947) and Percival (1947). Deans did not report the plant species classed as minor (less than 1% of pollen grains), which affected the overall species list found, and in

addition, 44% of samples contained pollen at important minor levels that could not be identified.

Supplementary References

1. Deans, A. S. C. *Survey of British Honey Sources*. (Bee Research Association, 1957).
2. Deans, A. S. C. The Pollen Analysis of some British Honeys. (Thesis for the National Bee Diploma, 1958).
3. Synge, A. D. Pollen collection by honeybees (*Apis mellifera*). *J. Anim. Ecol.* **16**, 122–138 (1947).
4. Percival, M. Pollen collection by *Apis mellifera*. *New Phytol.* **46**, 142–165 (1947).
5. Lucas, A. *et al.* Floral resource partitioning by individuals within generalised hoverfly pollination networks revealed by DNA metabarcoding. *Sci. Rep.* **8**, (2018).
6. de Vere, N. *et al.* DNA Barcoding the Native Flowering Plants and Conifers of Wales. *PLoS One* **7**, e37945 (2012).
