# Supplementary material for: Shifts in honeybee foraging reveal historical changes in floral resources
Source: Commun Biol. 2021 Jan 14;4:37. doi: 10.1038/s42003-020-01562-4 (PMC7809426; doi:10.1038/s42003-020-01562-4)
Supplement: Supplementary file 4 — Reporting Summary [file 42003_2020_1562_MOESM4_ESM.pdf]

nature research

Corresponding author(s): Natasha de Vere

Last updated by author(s): Nov 2, 2020

Reporting Summary

Nature Research wishes to improve the reproducibility of the work that we publish. This form provides structure for consistency and transparency in reporting. For further information on Nature Research policies, see Authors & Referees and the Editorial Policy Checklist .

Statistics

For all statistical analyses, confirm that the following items are present in the figure legend, table legend, main text, or Methods section.

n/a	Confirmed	
☐	☒	The exact sample size (*n*) for each experimental group/condition, given as a discrete number and unit of measurement
☐	☒	A statement on whether measurements were taken from distinct samples or whether the same sample was measured repeatedly
☐	☒	The statistical test(s) used AND whether they are one- or two-sided<br>*Only common tests should be described solely by name; describe more complex techniques in the Methods section.*
☐	☒	A description of all covariates tested
☐	☒	A description of any assumptions or corrections, such as tests of normality and adjustment for multiple comparisons
☒	☐	A full description of the statistical parameters including central tendency (e.g. means) or other basic estimates (e.g. regression coefficient) AND variation (e.g. standard deviation) or associated estimates of uncertainty (e.g. confidence intervals)
☐	☒	For null hypothesis testing, the test statistic (e.g. *F*, *t*, *r*) with confidence intervals, effect sizes, degrees of freedom and *P* value noted<br>*Give P values as exact values whenever suitable.*
☒	☐	For Bayesian analysis, information on the choice of priors and Markov chain Monte Carlo settings
☒	☐	For hierarchical and complex designs, identification of the appropriate level for tests and full reporting of outcomes
☐	☒	Estimates of effect sizes (e.g. Cohen's *d*, Pearson's *r*), indicating how they were calculated

Our web collection on statistics for biologists contains articles on many of the points above.

Software and code

Policy information about availability of computer code

Data collection	The Illumina MiSeq sequencing platform was used to sequence the honey samples from amplicon libraries. The sequencing was carried out at Liverpool Genomics, UK (https://www.liverpool.ac.uk/genomic-research/technologies/next-generation-sequencing/).
Data analysis	All quality filtering and merging of the paired end sequence data was carried out using a pipeline available on GitHub at: https://github.com/colford/nbgw-plant-illumina-pipeline (http://doi.org/10.5281/zenodo.4166248) and detailed in the methods section. Sequence data and metadata are available at the Sequence Read Archive, under the accession PRJNA577454. All statistical analyses of the quality filtered and identified sequence reads and GIS analyses were completed in R (v. 3.5.2). with the details of data treatment, and the functions and packages used detailed in the methods section.

For manuscripts utilizing custom algorithms or software that are central to the research but not yet described in published literature, software must be made available to editors/reviewers. We strongly encourage code deposition in a community repository (e.g. GitHub). See the Nature Research guidelines for submitting code & software for further information.

Data

Policy information about availability of data

All manuscripts must include a data availability statement. This statement should provide the following information, where applicable:

- Accession codes, unique identifiers, or web links for publicly available datasets
- A list of figures that have associated raw data
- A description of any restrictions on data availability

Sequence data and metadata are available at the Sequence Read Archive, under the accession PRJNA577454. Quality filtered and identified sequence data is available as supplementary material. This was used to generate Fig. 2-5, Supplementary Fig. 1-4 and 6-11 Fig. 1, Fig 3 and Supplementary Figure 5 were generated using metadata associated with the samples. Fig 1 and Fig 4 use data from the 2015 CEH Land Cover map data and 2017 CEH Land Cover plus: Crops which is available at https://doi.org/10.5285/6c6c9203-7333-4d96-88ab-78925e7a4e73. Specific location information for the sampled hives is not included in the supplementary data to protect the exact hive locations of the beekeepers. Location information with a precision to two decimal is included with the sequence metadata.

Field-specific reporting

Please select the one below that is the best fit for your research. If you are not sure, read the appropriate sections before making your selection.

☐ Life sciences ☐ Behavioural & social sciences ☒ Ecological, evolutionary & environmental sciences

For a reference copy of the document with all sections, see nature.com/documents/nr-reporting-summary-flat.pdf

Ecological, evolutionary & environmental sciences study design

All studies must disclose on these points even when the disclosure is negative.

Study description	This study analysed honeybee foraging using honey samples collected by beekeepers around the UK. In total, 441 honey samples were sequenced with two DNA barcode regions, rbcL and ITS2 in 2017. This data was compared with honey samples analysed using melissopalynology in 1952. Statistical models were used to test the effect of month of sampling and location (latitude and longitude) on honey plant composition, as well as examine differences between the two honey surveys.
Research sample	The research samples were honey samples collected by beekeepers in the UK from hives in 2017. The plants within the honey samples were characterised using two plant DNA barcode regions, rbcL and ITS2. Data from 1952 used honey samples collected by beekeepers in 1952 honey season and were characterised using melissopaynology.
Sampling strategy	Honey samples were requested from beekeepers across the UK from honey collected in 2017. Beekeepers were invited to supply honey for analysis via a nationwide campaign publicised on the gardening programme BBC Gardener's World (broadcast July 2017). Beekeepers were asked to sample the honey when they would normally remove the honey from the hives.
Data collection	Beekeepers sent in honey samples during the 2017 season, with completed survey forms noting the location and date of sampling. In total, 441 honey samples were sequenced with two DNA barcode regions, rbcL and ITS2. Data from the 1952 honey survey was collated from two sources, Deans, A. S. C. Survey of British Honey Sources. Bee Research Association, (1957) and Deans, A. S. C. The Pollen Analysis of some British Honeys. Thesis for the National Bee Diploma, (1958). Land cover data is accessible at: https://doi.org/10.5285/6c6c9203-7333-4d96-88ab-78925e7a4e73.
Timing and spatial scale	Beekeepers were asked to collect honey when they would normally remove the honey from the hives. Honey samples were collected from April to October with most samples provided in July (147 samples) and August (155 samples), representing the most likely time for beekeepers to extract honey from the hive in the UK. Samples were received from England, Wales, Scotland, Northern Ireland, and Ireland, with the majority of samples coming from England and Wales. Sample collection data and location information are available in the sequence metadata and supplementary material.
Data exclusions	Raw sequencing reads were trimmed to remove low quality regions, paired, and then merged, with merged reads shorter than 450 bp discarded. Identical reads were dereplicated within samples and then clustered at 100% identity across all samples, with singletons (sequence reads that occurred only once across all samples) discarded. Sequences that returned families from different clades were excluded. This retains high-quality accurate sequences and remove those with would lead to misidentification during taxonomic assignment. All sequencing data is available on the Sequence Read Archive SRA, accession number: PRJNA577454. All scripts used in analysis are available at: https://github.com/colford/nbgw-plant-illumina-pipeline. Samples which were successful with only one DNA barcode region were excluded from further analysis.
Reproducibility	This study involved sampling honey across the UK to investigate foraging patterns in the landscape. The reliability of the sequencing results were evaluated based on positive and negative controls.
Randomization	Honey samples were randomised on arrival for DNA extraction in 96-well plates.
Blinding	This study involved characterising honey across the UK to investigate foraging patterns in the UK. On arrival samples were given a unique identifying code without attached metadata. This code was used throughout sequence analysis in assigning taxonomic identifications, with location and sampling time only introduced during statistical analysis after taxonomic assignment.

Did the study involve field work? ☒ Yes ☐ No

Field work, collection and transport

Field conditions	Honey samples were collected by beekeepers throughout the foraging season (1/4/2017 to 10/10/2017) and sent to the National Botanic Garden of Wales in sterile tubes.
Location	Honey samples were collected across the UK, with the majority of sampling occurring in England and Wales. Sample locations are supplied in supplementary material and sequence metadata.
Access and import/export	Samples were collected by beekeepers with their agreement to participate in the study.
Disturbance	Samples were collected from managed honeybee hives when honey would normally be removed. No disturbance was caused during sampling.

Reporting for specific materials, systems and methods

We require information from authors about some types of materials, experimental systems and methods used in many studies. Here, indicate whether each material, system or method listed is relevant to your study. If you are not sure if a list item applies to your research, read the appropriate section before selecting a response.

Materials & experimental systems

n/a	Involved in the study
☒	☐ Antibodies
☒	☐ Eukaryotic cell lines
☒	☐ Palaeontology
☐	☒ Animals and other organisms
☒	☐ Human research participants
☒	☐ Clinical data

Methods

n/a	Involved in the study
☒	☐ ChIP-seq
☒	☐ Flow cytometry
☒	☐ MRI-based neuroimaging

Animals and other organisms

Policy information about studies involving animals; ARRIVE guidelines recommended for reporting animal research

Laboratory animals	Laboratory animals were not used in this study.
Wild animals	Honey was collected from managed hives by beekeepers when they would normally collect honey.
Field-collected samples	Honey was collected from managed hives by beekeepers when they would normally collect honey.
Ethics oversight	No ethical approval was required as honey was collected from managed hives by the beekeeper.

Note that full information on the approval of the study protocol must also be provided in the manuscript.
